# Notification that new names of prokaryotes, new combinations and new taxonomic opinions have appeared in volume 74, part 11 of the IJSEM

**DOI:** 10.1099/ijsem.0.006619

**Published:** 2025-03-03

**Authors:** Aharon Oren, Markus Göker

**Affiliations:** 1 The Institute of Life Sciences, The Hebrew University of Jerusalem, The Edmond J. Safra Campus, 9190401 Jerusalem, Israel; 2 Leibniz Institute DSMZ – German Collection of Microorganisms and Cell Cultures, Inhoffenstrasse 7B, 38124 Braunschweig, Germany

This listing of names of prokaryotes published in a previous issue of the *IJSEM* is provided as a service to microbiology to assist in the recognition of new names and new combinations. This procedure was proposed by the Judicial Commission [1]. The names given herein are listed according to the rules of priority (i.e. time and date of publication of the articles on the journal’s platform and the order of valid publication of names in the original articles) [2].

**Table:** 

Order of article publication	Name/authors	Proposed as	Article no.	Page no.
1	*Lactobacillus amylovorus* subsp. *animalium* Yamane *et al*. 2024	nom. nov. [replacement name for the illegitimate name *Lactobacillus amylovorus* subsp. *animalis* Yamane *et al*. 2024]	006564	1
2	*Microbispora maris* Xie *et al*. 2024	sp. nov.	006568	6
3	*Bifidobacterium favimelis* Li *et al*. 2024	sp. nov.	006573	7
4	*Roseovarius phycicola* Lee *et al*. 2024	sp. nov.	006574	8
4	*Roseovarius rhodophyticola* Lee *et al*. 2024	sp. nov.	006574	8
5	*Aurantiacibacter flavus* Park *et al*. 2024	sp. nov.	006578	5
5	*Aurantiacibacter gilvus* Park *et al*. 2024	sp. nov.	006578	9
6	*Olsenella absiana* Paek *et al*. 2024	sp. nov.	006579	6
7	*Campylobacter sputorum* subsp. *bovis* Miller *et al*. 2024*	subsp. nov.	006571	6
8	*Pseudomonas huanghezhanensis* Ge *et al*. 2024	sp. nov.	006584	10
8	*Pseudomonas fjordensis* Ge *et al*. 2024	sp. nov.	006584	11
9	*Dyadobacter helix* Lucena *et al*. 2024	sp. nov.	006570	9
9	*Dyadobacter linearis* Lucena *et al*. 2024	sp. nov.	006570	10
10	*Luteimonas flava* Lu *et al*. 2024	sp. nov.	006585	9
10	*Aquilutibacter* Lu *et al*. 2024	gen. nov.	006585	10
10	*Aquilutibacter rugosus* Lu *et al*. 2024	sp. nov.	006585	11
10	*Cognatiluteimonas* Lu *et al*. 2024	gen. nov.	006585	12
10	*Cognatiluteimonas lumbrici* (Cha *et al*. 2020) Lu *et al*. 2024	comb. nov. [basonym: *Luteimonas lumbrici* Cha *et al.* 2020]	006585	12
10	*Cognatiluteimonas weifangensis* Lu *et al*. 2024	sp. nov.	006585	12
10	*Cognatiluteimonas profundi* (Jin *et al*. 2020) Lu *et al*. 2024	comb. nov. [basonym: *Lysobacter profundi* Jin *et al*. 2020]	006585	14
10	*Cognatiluteimonas sedimenti* Lu *et al*. 2024	sp. nov.	006585	14
10	*Cognatiluteimonas telluris* Lu *et al*. 2024	sp. nov.	006585	14
10	*Noviluteimonas* Lu *et al*. 2024	gen. nov.	006585	14
10	*Noviluteimonas dokdonensis* (Oh *et al*. 2011) Lu *et al*. 2024	comb. nov. [basonym: *Lysobacter dokdonensis* Oh *et al*. 2011]	006585	14
10	*Noviluteimonas gilva* Lu *et al*. 2024	sp. nov.	006585	14
10	*Noviluteimonas caseinilytica* (Chhetri *et al*. 2020) Lu *et al*. 2024	comb. nov. [basonym: *Lysobacter caseinilyticus* Chhetri *et al*. 2020]	006585	14
10	*Noviluteimonas lactosilytica* Lu *et al*. 2024	sp. nov.	006585	15
10	*Pseudoluteimonas* Lu *et al*. 2024	gen. nov.	006585	15
10	*Pseudoluteimonas lycopersici* (Lin *et al*. 2015) Lu *et al*. 2024	comb. nov. [basonym: *Lysobacter lycopersici* Lin *et al*. 2015]	006585	15
10	*Solilutibacter* Lu *et al*. 2024	gen. nov.	006585	15
10	*Solilutibacter tolerans* (Rani *et al*. 2016) Lu *et al*. 2024	comb. nov. [basonym: *Luteimonas tolerans* Rani *et al*. 2016]	006585	15
10	*Solilutibacter oculi* Lu *et al*. 2024	sp. nov.	006585	15
10	*Solilutibacter pythonis* (Busse *et al*. 2020) Lu *et al*. 2024	comb. nov. [basonym: *Lysobacter pythonis* Busse *et al*. 2020]	006585	16
10	*Solilutibacter silvestris* (Margesin *et al*. 2018) Lu *et al*. 2024	comb. nov. [basonym: *Lysobacter silvestris* Margesin *et al*. 2018]	006585	16
10	*Agrilutibacter* Lu *et al*. 2024	gen. nov.	006585	16
10	*Agrilutibacter niabensis* (Weon *et al*. 2007) Lu *et al*. 2024	comb. nov. [basonym: *Lysobacter niabensis* Weon *et al*. 2007]	006585	16
10	*Agrilutibacter solisilvae* Lu *et al*. 2024	sp. nov.	006585	16
10	*Agrilutibacter terrestris* (Woo and Kim 2022) Lu *et al*. 2024	comb. nov. [basonym: *Lysobacter terrestris* Woo and Kim 2022]	006585	16
10	*Cognatilysobacter* Lu *et al*. 2024	gen. nov.	006585	16
10	*Cognatilysobacter xinjiangensis* (Liu *et al*. 2011) Lu *et al*. 2024	comb. nov. [basonym: *Lysobacter xinjiangensis* Liu *et al*. 2011]	006585	17
10	*Cognatilysobacter bugurensis* (Zhang *et al*. 2011) Lu *et al*. 2024	comb. nov. [basonym: *Lysobacter bugurensis* Zhang *et al*. 2011]	006585	17
10	*Cognatilysobacter lacus* (Im *et al*. 2020) Lu *et al*. 2024	comb. nov. [basonym: *Lysobacter lacus* Im *et al*. 2020]	006585	17
10	*Cognatilysobacter segetis* (Ten *et al*. 2020) Lu *et al*. 2024	comb. nov. [basonym: *Lysobacter segetis* Ten *et al*. 2020]	006585	17
10	*Cognatilysobacter tabacisoli* (Xiao *et al*. 2019) Lu *et al*. 2024	comb. nov. [basonym: *Lysobacter tabacisoli* Xiao *et al*. 2019]	006585	17
10	*Cognatilysobacter terrigena* (Li *et al*. 2020) Lu *et al*. 2024	comb. nov. [basonym: *Lysobacter terrigena* Li *et al*. 2020]	006585	18
10	*Marilutibacter* Lu *et al*. 2024	gen. nov.	006585	18
10	*Marilutibacter maris* (Yoon 2016) Lu *et al*. 2024	comb. nov. [basonym: *Lysobacter maris* Yoon 2016]	006585	18
10	*Marilutibacter aestuarii* (Jeong *et al*. 2016) Lu *et al*. 2024	comb. nov. [basonym: *Lysobacter aestuarii* Jeong *et al*. 2016]	006585	18
10	*Marilutibacter alkalisoli* (Xu *et al*. 2020) Lu *et al*. 2024	comb. nov. [basonym: *Lysobacter alkalisoli* Xu *et al*. 2020]	006585	18
10	*Marilutibacter chinensis* Lu *et al*. 2024	sp. nov.	006585	18
10	*Marilutibacter penaei* (Xu *et al*. 2021) Lu *et al*. 2024	comb. nov. [basonym: *Lysobacter penaei* Xu *et al*. 2021]	006585	19
10	*Marilutibacter spongiae* (Choi *et al*. 2019) Lu *et al*. 2024	comb. nov. [basonym: *Lysobacter spongiae* Choi *et al*. 2019]	006585	19
10	*Novilysobacter* Lu *et al*. 2024	gen. nov.	006585	19
10	*Novilysobacter concretionis* (Bae *et al*. 2005) Lu *et al*. 2024	comb. nov. [basonym: *Lysobacter concretionis* Bae *et al*. 2005]	006585	19
10	*Novilysobacter antarcticus* (Liu *et al*. 2022) Lu *et al*. 2024	comb. nov. [basonym: *Lysobacter antarcticus* Liu *et al*. 2022]	006585	19
10	*Novilysobacter arseniciresistens* (Luo *et al*. 2012) Lu *et al*. 2024	comb. nov. [basonym: *Lysobacter arseniciresistens* Luo *et al*. 2012]	006585	19
10	*Novilysobacter avium* (Lee *et al*. 2022) Lu *et al*. 2024	comb. nov. [basonym: *Lysobacter avium* Lee *et al*. 2022]	006585	20
10	*Novilysobacter ciconiae* (Lee *et al*. 2022) Lu *et al*. 2024	comb. nov. [basonym: *Lysobacter ciconiae* Lee *et al*. 2022]	006585	20
10	*Novilysobacter defluvii* (Yassin *et al*. 2007) Lu *et al*. 2024	comb. nov. [basonym: *Lysobacter defluvii* Yassin *et al*. 2007]	006585	20
10	*Novilysobacter erysipheiresistens* (Xie *et al*. 2016) Lu *et al*. 2024	comb. nov. [basonym: *Lysobacter erysipheiresistens* Xie *et al*. 2016]	006585	20
10	*Novilysobacter luteus* (Lucena *et al*. 2022) Lu *et al*. 2024	comb. nov. [basonym: *Lysobacter luteus* Lucena *et al*. 2022]	006585	20
10	*Novilysobacter selenitireducens* (Mao *et al*. 2022) Lu *et al*. 2024	comb. nov. [basonym: *Lysobacter selenitireducens* Mao *et al*. 2022]	006585	20
10	*Novilysobacter spongiicola* (Romanenko *et al*. 2008) Lu *et al*. 2024	comb. nov. [basonym: *Lysobacter spongiicola* Romanenko *et al*. 2008]	006585	21
10	*Montanilutibacter* Lu *et al*. 2024	gen. nov.	006585	21
10	*Montanilutibacter psychrotolerans* (Luo *et al*. 2019) Lu *et al*. 2024	comb. nov. [basonym: *Lysobacter psychrotolerans* Luo *et al*. 2019]	006585	21
10	*Aerolutibacter* Lu *et al*. 2024	gen. nov.	006585	21
10	*Aerolutibacter daejeonensis* (Weon *et al*. 2006) Lu *et al*. 2024	comb. nov. [basonym: *Lysobacter daejeonensis* Weon *et al*. 2006]	006585	21
10	*Aerolutibacter ruishenii* (Wang *et al*. 2011) Lu *et al*. 2024	comb. nov. [basonym: *Lysobacter ruishenii* Wang *et al*. 2011]	006585	22
11	*Aeribacillus alveayuensis* (Bae *et al*. 2005) Li *et al*. 2024	comb. nov. [basonym: *Bacillus alveayuensis* Bae *et al*. 2005]	006539	18
11	*Aeribacillus kexueae* (Sun *et al*. 2018) Li *et al*. 2024	comb. nov. [basonym: *Bacillus kexueae* Sun *et al*. 2018]	006539	18
11	*Alkalihalobacterium alkalicellulosilyticum* corrig. (Liu *et al*. 2022) Li *et al*. 2024†	comb. nov. [basonym: *Bacillus alkalicellulosilyticus* Liu *et al*. 2022]	006539	26
11	*Halalkalibacter suaedae* (Lei *et al*. 2022) Li *et al*. 2024	comb. nov. [basonym: *Bacillus suaedae* Lei *et al*. 2022]	006539	27
11	*Anoxybacillus andreesenii* (Kosowski *et al*. 2014) Li *et al*. 2024‡	comb. nov. [basonym: *Bacillus andreesenii* Kosowski *et al*. 2014]	006539	28
11	*Aureibacillaceae* Li *et al*. 2024	fam. nov.	006539	29
11	*Caldalkalibacillus horti* (Yumoto *et al*. 1998) Li *et al*. 2024	comb. nov. [basonym: *Bacillus horti* Yumoto *et al*. 1998]	006539	31
11	*Ureibacillus composti* (Hayat *et al*. 2014) Li *et al*. 2024	comb. nov. [basonym: *Lysinibacillus composti* Hayat *et al*. 2014]	006539	34
11	*Ureibacillus endophyticus* (Yu *et al*. 2017) Li *et al*. 2024	comb. nov. [basonym: *Lysinibacillus endophyticus* Yu *et al*. 2017]	006539	35
11	*Ureibacillus yapensis* (Yu *et al*. 2020) Li *et al*. 2024	comb. nov. [basonym: *Lysinibacillus yapensis* Yu *et al*. 2020]	006539	35
11	*Cytobacillaceae* Li *et al*. 2024	fam. nov.	006539	35
11	*Domibacillaceae* Li *et al*. 2024	fam. nov.	006539	36
11	*Falsibacillaceae* Li *et al*. 2024	fam. nov.	006539	38
11	*Heyndrickxiaceae* Li *et al*. 2024	fam. nov.	006539	39
11	*Lottiidibacillaceae* Li *et al*. 2024	fam. nov.	006539	41
11	*Oxalophagaceae* Li *et al*. 2024	fam. nov.	006539	43
11	*Pradoshiaceae* Li *et al*. 2024	fam. nov.	006539	46
11	*Rossellomoreaceae* Li *et al*. 2024	fam. nov.	006539	47
11	*Rossellomorea pakistanensis* (Roohi *et al*. 2014) Li *et al*. 2024	comb. nov. [basonym: *Bacillus pakistanensis* Roohi *et al*. 2014]	006539	47
11	*Schinkiaceae* Li *et al*. 2024	fam. nov.	006539	50
11	*Sulfoacidibacillaceae* Li *et al*. 2024	fam. nov.	006539	52
11	*Sutcliffiellaceae* Li *et al*. 2024	fam. nov.	006539	52
11	*Litchfieldia luteola* (Shi *et al*. 2011) Li *et al*. 2024	comb. nov. [basonym: *Bacillus luteolus* Shi *et al*. 2011]	006539	53
11	*Litchfieldia sinesaloumensis* (Sarr *et al*. 2021) Li *et al*. 2024	comb. nov. [basonym: *Bacillus sinesaloumensis* Sarr *et al*. 2021]	006539	53
11	*Litchfieldia suaedae* (Xu *et al*. 2022) Li *et al*. 2024	comb. nov. [basonym: *Cytobacillus suaedae* Xu *et al*. 2022]	006539	53
11	*Priestia iocasae* (Wang *et al*. 2017) Li *et al*. 2024	comb. nov. [basonym: *Bacillus iocasae* Wang *et al*. 2017]	006539	53
11	*Sutcliffiella tianshenii* (Jiang *et al*. 2014) Li *et al*. 2024	comb. nov. [basonym: *Bacillus tianshenii* Jiang *et al*. 2014]	006539	54
12	*Clostridium lapidicellarium* Yang *et al*. 2024	sp. nov.	006580	6
12	*Clostridium moutaii* Yang *et al*. 2024	sp. nov.	006580	7
13	*Roseicyclus sediminis* Zhou *et al*. 2024	sp. nov.	006583	6
13	*Roseicyclus salinarum* Zhou *et al*. 2024	sp. nov.	006583	6
13	*Roseicyclus elongatus* (Suzuki *et al*. 2006) Zhou *et al*. 2024	comb. nov. [basonym: *Roseibacterium elongatum* Suzuki *et al*. 2006]	006583	8
13	*Roseicyclus persicicus* (Yoon 2020) Zhou *et al*. 2024	comb. nov. [basonym: *Roseibacterium persicicum* Yoon 2020]	006583	8
14	*Alterisphingorhabdus* Yan *et al*. 2024	gen. nov.	006577	8
14	*Alterisphingorhabdus coralli* Yan *et al*. 2024	sp. nov.	006577	8
15	*Blastococcus montanus* Wang *et al*. 2024	sp. nov.	006546	9
16	*Pseudomonas machongensis* Qiu *et al*. 2024	sp. nov.	006550	8
16	*Stenotrophomonas capsici* Qiu *et al*. 2024	sp. nov.	006550	8
17	*Plantactinospora soli* Wang *et al*. 2024	sp. nov.	006590	9
18	*Kosakonia calanthes* Wu *et al*. 2024	sp. nov.	006594	6
19	*Rothia uropygioeca* (Braun *et al*. 2018) Ghodhbane-Gtari *et al*. 2024¶	comb. nov. [basonym: *Kocuria uropygioeca* Braun *et al*. 2018]	006586	7
19	*Rothia uropygialis* (Braun *et al*. 2018) Ghodhbane-Gtari *et al*. 2024	comb. nov. [basonym: *Kocuria uropygialis* Braun *et al*. 2018]	006586	7
19	*Kocuria marina* subsp. *marina* (Kim *et al*. 2004) Ghodhbane-Gtari *et al*. 2024	comb. nov. [basonym: *Kocuria marina* Kim *et al*. 2004]	006586	7
19	*Kocuria marina* subsp. *indica* (Dastager *et al*. 2014) Ghodhbane-Gtari *et al*. 2024	comb. nov. [basonym: *Kocuria indica* Dastager *et al*. 2014]	006586	7
19	*Kocuria rosea* subsp. *rosea* (Flügge 1886) Ghodhbane-Gtari *et al*. 2024	comb. nov. [basonym: *Micrococcus roseus* Flügge 1886 (Approved Lists 1980)]	006586	7
19	*Kocuria rosea* subsp. *polaris* (Reddy *et al*. 2003) Ghodhbane-Gtari *et al*. 2024	comb. nov. [basonym: *Kocuria polaris* Reddy *et al*. 2003]	006586	7

*The authors also described *Campylobacter sputorum* subsp. *sputorum* as subsp. nov., but the name *Campylobacter sputorum* subsp. *sputorum* (Prévot 1940) Véron and Chatelain 1973 (Approved Lists 1980) was already validly published.

†The etymology should read N.L. neut. adj. *alkalicellulosilyticum*, alkaline cellulose dissolving instead of N.L. masc. adj. …

‡The protologue text gives *Bacillus andreesenii* instead of *Anoxybacillus andreesenii*.

¶Spelled *Rothia uropygioeae* in the protologue heading.

The following taxonomic opinions (i.e. the emendation of circumscriptions or the creation of synonyms) do not affect the status of a name under the International Code of Nomenclature of Prokaryotes. These taxonomic opinions can neither be considered as approved nor as disapproved by the International Committee on Systematics of Prokaryotes.

**Table:** 

Name/authors:	Article no.	Page no.
*Aeribacillaceae* Chuvochina *et al*. 2023 emend. Li *et al*. 2024	006539	12
*Alicyclobacillaceae* Costa and Rainey 2010 emend. Li *et al*. 2024	006539	19
*Amphibacillaceae* Chuvochina *et al*. 2023 emend. Li *et al*. 2024	006539	19
*Anaerobacillaceae* Chuvochina *et al*. 2023 emend. Li *et al*. 2024	006539	25
*Anoxybacillaceae* Chuvochina *et al*. 2023 emend. Li *et al*. 2024	006539	28
*Bacillaceae* Fischer 1895 (Approved Lists 1980) emend. Li *et al*. 2024	006539	29
*Caldalkalibacillaceae* Chuvochina *et al*. 2023 emend. Li *et al*. 2024	006539	30
*Caldibacillaceae* Chuvochina *et al*. 2023 emend. Li *et al*. 2024	006539	31
*Calditerricolaceae* Chuvochina *et al*. 2023 emend. Li *et al*. 2024	006539	31
*Campylobacter sputorum* (Prévot 1940) Véron and Chatelain 1973 (Approved Lists 1980) emend. Miller *et al*. 2024	006571	6
*Caryophanaceae* Peshkoff 1939 (Approved Lists 1980) emend. Li *et al*. 2024	006539	31
*Effusibacillaceae* Chuvochina *et al*. 2023 emend. Li *et al*. 2024	006539	37
*Exiguobacteriaceae* Chuvochina *et al*. 2023 emend. Li *et al*. 2024	006539	38
*Fictibacillaceae* Chuvochina *et al*. 2023 emend. Li *et al*. 2024	006539	38
*Jeotgalibacillaceae* Chuvochina *et al*. 2023 emend. Li *et al*. 2024	006539	40
*Kocuria marina* Kim *et al*. 2004 emend. Ghodhbane-Gtari *et al*. 2024	006586	7
*Kocuria rosea* Stackebrandt *et al*. 1995 emend. Ghodhbane-Gtari *et al*. 2024	006586	7
*Kyrpidiaceae* Chuvochina *et al*. 2023 emend. Li *et al*. 2024	006539	40
*Marinococcaceae* Chuvochina *et al*. 2023 emend. Li *et al*. 2024	006539	41
*Olsenella* Kaatz *et al.* 20211 emend. Paek *et al.* 2024	006579	7
*Paenibacillaceae* De Vos *et al.* 2010 emend. Li *et al.* 2024	006539	44
*Salisediminibacteriaceae* Chuvochina *et al.* 2023 emend. Li *et al.* 2024	006539	48
*Spiroplasma atrichopogonis* Koerber *et al.* 2005 pro synon. *Spiroplasma mirum* Tully *et al.* 1982	006589	4
*Sporolactobacillaceae* Ludwig *et al.* 2010 emend. Li *et al.* 2024	006539	50
*Thermicanaceae* Chuvochina *et al.* 2024 emend. Li *et al.* 2024	006539	54
*Thermoactinomycetaceae* Matsuo *et al.* 2006 emend. Li *et al.* 2024	006539	54
